# Global impact of vaccine nationalism during COVID-19 pandemic

**DOI:** 10.1186/s41182-021-00394-0

**Published:** 2021-12-29

**Authors:** Mehr Muhammad Adeel Riaz, Unaiza Ahmad, Anmol Mohan, Ana Carla dos Santos Costa, Hiba Khan, Maryam Salma Babar, Mohammad Mehedi Hasan, Mohammad Yasir Essar, Ahsan Zil-E-Ali

**Affiliations:** 1 Punjab Medical College, Faisalabad, Pakistan; 2 Karachi Medical & Dental College, Karachi, Pakistan; 3 Federal University of Bahia, Salvador, Bahia Brazil; 4 Dubai Health Authority, Dubai, United Arab Emirates; 5 Department of Biochemistry and Molecular Biology, Faculty of Life Science, Mawlana Bhashani Science and Technology University, Tangail, Bangladesh; 6 Kabul University of Medical Sciences, 1001 Kabul, Afghanistan; 7 Pennsylvania State University, State College, PA USA

**Keywords:** Vaccine, Nationalism, COVID-19, Impact, Vaccine nationalism

## Abstract

Vaccines are the best chance to control the pandemic—unless leaders succumb to vaccine nationalism. Vaccine nationalism is a frequent recurrence, especially during a brand-new market distribution. The development of safe and effective COVID-19 vaccines in such a short space of time is a testament to modern scientific abilities. It will also test the world's political will and moral commitment to end this pandemic. As desperate as the COVID-19 pandemic, vaccine nationalism is already setting a foundation for itself and is considered socially and economically counterproductive. Vaccine equity is not just a theoretical slogan, and it protects people worldwide from new vaccine-resistant variants. Understanding and anticipating the consequences is vital, and creating a global solution approach to avoid them. This article evaluates the common issues previously faced and the plausible ones during this pandemic. A few recommendations are made to warn and accentuate the reality of this dire matter.

## Introduction

Vaccine nationalism is an economic strategy to hoard vaccinations from manufacturers and increase supply in their own country. The aim is to stock up and vaccinate the nation as soon as possible regardless of the limited vaccine manufacturers' distribution for the rest of the world. High-income countries (HICs) put their money in beforehand for many vaccine stocks, which hinders the manufacturer's ability to supply globally. The vaccines that wealthy countries pay for are enough to vaccinate their whole population twice or thrice, manifesting it against the global promises of equitable supply of vaccines among low-and-middle-income countries (LMICs), thus establishing the bias towards the population of Global South [1].

It leaves unprivileged countries of the ‘Global South’ with a lower economic status to struggle in paying dues for vaccinating their population as the prices inflate over the time. It also decreases the opportunity for developing countries to access supplies as they would be relatively out of stock [2] Thus, the risk of this recurring appears to be favorable with the crashing economies and exacerbating death rate [2]

## Vaccine availability against SARS-CoV-2

The sudden onset and worsening progress of the SARS-CoV-2 pandemic motivated the multilateral global organizations and individual private funders to collectively fund research for an effective vaccine against the novel coronavirus. Large number of scientific personnel, technicians and data managers are involved from the laboratory-based experimentation to conducting clinical trials across the globe. Only 18 vaccines were listed as candidates for assessment for WHO emergency use listing and prequalification as of November 1, 2021 [3]. However, arguing about the production of more vaccine candidates and their efficiencies would be out of the scope of this article as every year, new vaccines enter clinical trials. They are made available in the open market based on established efficiencies and safety. Nevertheless, the pertinent issue here is that once the vaccine is available in the market for public consumption, it should be distributed equitably among the nations without any necessity to negotiate its supply with high-income countries, governments, and relevant stakeholders, such as multilateral organizations and pharmaceuticals. The irony associated with this situation is that high-income countries decided to invest in the stock of the vaccinations instead of spending that money for immediate capacity building and delivery of life-saving drugs by healthcare systems [4].

One of the fundamental factors in understanding vaccine nationalism is that many pharmaceutical companies from different countries competed in vaccine production. However, it was not to “aid the ailing humanity” at “minimum cost” but to sell their product to other countries for a “strong financial stock upholds” [2]. According to data analytics, most vaccine doses in 2020 and 2021 were estimated to be produced in the United States (4.69 billion), India (3.13 billion), China (1.90 billion), United Kingdom (0.95 billion), Germany (0.50 billion) and South Korea (0.35 billion) [5]. Each pharmaceutical company had its agreements to sell their vaccine depending on the estimated profit while excluding the requirement to distribute towards impoverished countries. This led to devastating results for the LMICs left behind in the global race of immunization against the deadly SARS-CoV-2. Many studies also support that vaccine inequity during the pandemic creates a considerable barrier to attaining global immunity [6]. Therefore, it can disturb all efforts in ending the COVID-19 mortalities and intervene in economic recovery, which collapsed drastically during the pandemic [7].

## History of vaccine nationalism

Vaccine nationalism is not a new phenomenon and has been maliciously practiced by several high-income nations during previous health crises [8]. The prioritization of one's population is understandable; however, the method by which it has been achieved is deemed morally and ethically deplorable. The human right to health is expressed in many international declarations and conventions, including Article 5 of the Convention on eliminating all forms of racial discrimination. In the debate on the right to health, Article 12 of the International Covenant on Economic Social and Cultural Rights (ICESCR) is the main focus [9]. ICESCR seemingly acknowledges human rights on behalf of all members of the human family, but it is noted that the language in the ICESCR lacks any jurisdictional restrictions. Unlike other corresponding international agreements, ICESCR has made it easier for countries to exercise unethical conduct to secure vaccines in enormous quantities while developing countries struggle to attain them at all [10].

For instance, in 2009, the feared limited production of the H1N1 vaccine affected the availability of the doses of vaccine in developing countries [8]. The Australian government ensured that their local manufacturer, the Commonwealth Serum Laboratories, would meet the government's domestic demands prior to shipping the vaccines to the United Nations [11]. This pattern has also been observed in initial vaccination distribution for polio and smallpox [12].

## Impact of vaccine nationalism

Another adverse effect is the ability of vaccine nationalism to stretch the duration of the pandemic further. Consequently, there will be an increase in the disease's adverse outcomes and the overload of already overburdened health systems. It was forecasted that more than half of the vaccine doses produced in 2021; have been purchased in advance by high-income countries even though they make up 13 percent of the world's population [13]. This highlights the disturbing difference between the 39 million doses in high-income nations and the 25 doses in low-income countries [14]. Italy banned the AstraZeneca vaccine exports, another example of a high-income country trying to hoard vaccines for their citizens [15].

Vaccine stockpiling will eventually be self-defeating. As evident, although most vaccine doses were administered in high-income countries, according to the study conducted by researchers based in Oxford, 27 of the high-income countries such as Italy, France, and the UK reported all most 1 million extra deaths than projected by epidemiologists [16]. Better medical infrastructure, i.e., availability and accessibility of rapid COVID-19 testing, data reporting transparency, and prevalence of underlying diseases (diabetes, hypertension, and ischemic heart diseases, etc.) coupled with the prevalence of the majority of the elderly population and geographical conditions can be the cause of more deaths in high-income countries [16]. Earlier this year, Brazil and Bangladesh reported new COVID-19 variants. That turned the country into a breeding ground for it and emerging viruses, such as Zika that still threaten the globe [17, 18]. As the new variant continues to spread, it is clear that vaccinating one country's population would not provide long-term immunity, mutating into possibly vaccine-resistant forms. Moreover, the increase in the number of infected people can provide a favorable environment for further mutations of the virus [19].

Consequently, variants with greater transmissibility and severity, such as the Delta variant and Omicron variant, are more challenging to identify by existing diagnostic tests and are resistant to many therapies, such as monoclonal antibodies. They can escape natural or vaccine-induced immunity to eventually become a dominant strain that attacks previously vaccinated individuals of the old strain [20]. Although it remains unclear whether mutations make vaccines less effective in preventing COVID-19, research has shown that antibodies produced by the Pfizer/BioNTech vaccine were less effective in neutralizing the study’s sample virus, which is similar to variant B.1.351, compared to the strain without these mutations [21]. Pharmaceutical companies have stated that they can adapt vaccines to fight new emerging variants; however, it is uncertain how long this process would take to happen and the logistics employed in the revaccination campaigns [22].

To control the pandemic, it is necessary to create COVID-19 communal immunity, thus reducing the virus's ability to transmit across populations. A large section of the population must be vaccinated against the disease to achieve this. The WHO estimates that the pandemic's effects may not subside until a significant section of the global total is vaccinated. Therefore, vaccine nationalism will ultimately prolong this pandemic, as the estimations show that low-income countries will not get access to the vaccine they need before 2023–2024 [13, 23].

Due to globalization, developed countries depend on commercial partners from developing countries to maintain their economies. The predictions are that the pandemic could result in losses of US$ 1 trillion per year, giving a total of US$ 119 billion in high-income countries, such as the United States. In contrast, the cost of providing vaccines to low-income countries is estimated to be approximately U$ 25 billion [22].

The pandemic will exacerbate already fragile health systems and economies in underdeveloped and developing countries. According to the World Bank, it is expected that, worldwide, extreme poverty will increase for the first time in 20 years and that around 150 million people will live in conditions of extreme poverty, with less than US$ 1.90 per day. Therefore, vaccine nationalism threatens to exacerbate extreme poverty by triggering greater income inequality and social immobility vulnerable [24].

## Recommendations and conclusion

In conclusion, the long-term adverse effect of vaccine nationalism cannot be ignored as the global impact is severe even if it supposedly protects individual nations. Being willing to cooperate could solve the global crisis, the common goal. The temptation for all the governments to concentrate on safeguarding their people should be overcome instead of jeopardizing the long-term plan. Vaccines should be accessible to everyone irrespective of their location and nationality.

To tackle vaccine nationalism, it is essential to develop a science-based strategy and mobilize an international collaboration and collective effort. The majority of the global policymakers have limited role in managing health economics, and not directly involved in clinical aspects of countries’ healthcare system; instead, the inflow of capital is controlled by the finance ministries and disbursed by health ministries. This disjoint between the funds, and allocation of the resources are misleading. Thus, the root of the problem is the supply versus demand mismatch in COVID-19 vaccines, which is an economic issue, NOT a health issue. Efforts for increasing global production and supply without sacrificing their efficacy and safety should be the first step towards eliminating vaccine nationalism. Moreover, epidemiologists, virologists, and social scientists should work with governments of all countries to formulate an action plan aimed towards rapid vaccination of high-risk individuals, including healthcare professionals and patients with comorbidities. Consequently, allowing a faster approach to herd immunity instead of prolonging the pandemic by increasing vaccine nationalism. A more inclusive approach is required to establish vaccine equity. If this is not achieved, the consequences of vaccine nationalism will be far beyond the borders and can lead to irreparable damage to the suffering societies. Finding a solution to vaccine nationalism is a matter of adjusting the moral compass and supporting justice. While there may not be a straightforward solution, it is definitely not an impossible pursuit to back vaccine equity for all the nations.

## Data Availability

Not applicable.

## Abbreviations

COVID-19: Coronavirus disease 2019
SARS-CoV-2: Severe acute respiratory syndrome coronavirus 2
LMICs: Low and middle-income countries
WHO: World Health Organization
ICESCR: International Covenant on Economic Social and Cultural Rights

## References

[1] World Economic Forum. Coronavirus: What is vaccine nationalism, how it affects us? (n.d.). https://www.weforum.org/agenda/2021/01/what-is-vaccine-nationalism-coronavirus-its-affects-covid-19-pandemic/. Accessed 01 Nov 2021.

[2] Cem Çakmaklı, Selva Demiralp, Sebnem Kalemli-Ozcan, Sevcan Yeşiltaş, Muhammed A. Yıldırım. The economic case for global vaccination: an Epidemiological Model with International Production Networks https://iccwbo.org/content/uploads/sites/3/2021/02/2021-icc-summary-for-policymakers.pdf. Accessed 01 Nov 2021.

[3] World Health Organization (WHO). Status of COVID-19 Vaccines within WHO EUL/PQ evaluation process, n.d. https://extranet.who.int/pqweb/sites/default/files/documents/Status_COVID_VAX_01March2021.pdf. Accessed 01 Nov 2021.

[4] David E. Bloom, Daniel Cadarette, and Daniel L. Tortorice. Our approach to vaccine finance is ill-suited to addressing epidemic risk. https://www.imf.org/external/pubs/ft/fandd/2020/09/vaccine-finance-epidemics-and-prevention-bloom.htm. Accessed 01 Nov 2021.

[5] A. Buchholz, Where Coronavirus Vaccines Will Be Produced, Statista. (2021). https://www.statista.com/chart/23885/coronavirus-vaccine-production-capabilities-by-country/. Accessed 01 Nov 2021.

[6] Stephenson J (2021). Unequal access to COVID-19 vaccines leaves less-wealthy countries more vulnerable, poses threat to global immunity. JAMA Health Forum.

[7] Duan Y, Shi J, Wang Z, Zhou S, Jin Y, Zheng ZJ (2021). Disparities in COVID-19 vaccination among low-, middle-, and high-income countries: the mediating role of vaccination policy. Vaccines.

[8] Institute of Medicine (US) Forum on Medical and Public Health Preparedness for Catastrophic Events. The 2009 H1N1 Influenza Vaccination Campaign: Summary of a Workshop Series. Washington (DC): National Academies Press (US); 2010. 2, Vaccine Supply. https://www.ncbi.nlm.nih.gov/books/NBK54181/. Accessed 01 Nov 2021. 21595118

[9] The Office of the High Commissioner for Human Rights (OHCHR). International Covenant on Economic, Social and Cultural Rights, (n.d.). https://www.ohchr.org/EN/ProfessionalInterest/Pages/CESCR.aspx. Accessed 01 Nov 2021.

[10] A.P.R. Oona Hathaway, Preston Lim, Mark Stevens, COVID-19 and International Law Series: Introduction, Just Secur. (2020). https://www.justsecurity.org/73304/covid-19-and-international-law-series-introduction/. Accessed 01 Nov 2021.

[11] Fidler DP (2010). Negotiating equitable access to influenza vaccines: global health diplomacy and the controversies surrounding avian influenza H5N1 and pandemic influenza H1N1. PLoS Med.

[12] Fidl DP (2020). Vaccine nationalism’s politics. Science.

[13] Mullard A (2020). How COVID vaccines are being divied up around the world. Nature.

[14] WHO: just 25 Covid vaccine doses administered in low-income countries | Vaccines and immunisation | The Guardian, (n.d.). https://www.theguardian.com/society/2021/jan/18/who-just-25-covid-vaccine-doses-administered-in-low-income-countries. Accessed 01 Nov 2021.

[15] T. John, Italy blocks AstraZeneca vaccines as fears grow over vaccine nationalism, CNN. (2021). https://edition.cnn.com/2021/03/05/world/coronavirus-newsletter-03-05-21-int/index.html. Accessed 01 Nov 2021.

[16] Bayati M (2021). Why is COVID-19 more concentrated in countries with high economic status?. Iran J Public Health.

[17] dos Santos Costa AC, Hasan MM, Xenophontos E, Mohanan P, Bassey EE, Hashim HT, Ahmad S, Essar MY (2021). COVID-19 and Zika: an emerging dilemma for Brazil. J Med Virol.

[18] Hasan MM, Rocha ICN, Ramos KG, Cedeño TDD, dos Santos Costa AC, Tsagkaris C, Billah MM, Ahmad S, Essar MY (2021). Emergence of highly infectious SARS-CoV-2 variants in Bangladesh: the need for systematic genetic surveillance as a public health strategy. Trop Med Heal.

[19] M.V.B. Andre Romani Pinto, Simone Preissler Iglesias, World’s worst covid crisis is unfolding in brazil, where no fix seems to work. (2021). https://www.bloomberg.com/news/features/2021-03-05/coronavirus-crisis-in-brazil-deepens-as-covid-variant-spreads-and-deaths-spike.

[20] The Centers for Disease Control and Prevention (CDC). Surveillance for SARS-CoV-2 Variants, (n.d.). https://www.cdc.gov/coronavirus/2019-ncov/cases-updates/variant-surveillance.html. Accessed 01 Nov 2021.

[21] Liu Y, Liu J, Xia H, Zhang X, Fontes-Garfias CR, Swanson KA, Cai H, Sarkar R, Chen W, Cutler M, Cooper D, Weaver SC, Muik A, Sahin U, Jansen KU, Xie X, Dormitzer PR, Shi P-Y (2021). Neutralizing activity of BNT162b2-elicited serum. N Engl J Med.

[22] Hafner M, Yerushalmi E, Fays C, Dufresne E, Van Stolk C (2020). COVID-19 and the cost of vaccine nationalism. RAND Corp.

[23] World Health Organization (WHO). Coronavirus disease (COVID-19): Herd immunity, lockdowns and COVID-19, (n.d.). https://www.who.int/news-room/q-a-detail/herd-immunity-lockdowns-and-covid-19. Accessed 01 Nov 2021.

[24] The World Bank. COVID-19 to Add as Many as 150 Million Extreme Poor by 2021, 2020. (n.d.). https://www.worldbank.org/en/news/press-release/2020/10/07/covid-19-to-add-as-many-as-150-million-extreme-poor-by-2021. Accessed 01 Nov 2021.

